# Medical Items

**Published:** 1918-02

**Authors:** 


### MEDICAL ITEMS.

### CAMPHOR IN OIL IN PNEUMONIA.

Camphor when added to culture media even in the proportion of 1 to 10,000 inhibits the growth of pneumococci. A series of experiments on rabbits n which an emulsion of pneumococci was injected intravenously, showed that injections of camphorated oil retarded death from two to five days in all and in 50 per cent of the cases prevented it. Clinical experience has demonstrated that hypodermatic injections of camphor are not toxic. A prominent physician of New York City has given over four thousand injections of camphor in oil sometimes giving as high as one hundred and fifty grains daily to one patient without any symptons of poisoning.

Camphor hypodermatically exerts an inhibitory action on the pneumocci in the blood stream and appears to have an antitoxic effect analogous to diphtheria antitoxin.

It is recommended that a dose of 10 units be injected as soon as possible after the initial chill and repeated every night hours except in bilateral pneumonia and in case with severe toxemia in which injections of fifteen to twenty mils should be given every six to eight hours. When temperature, pulse and respiration become normal, injections can be made every twenty-four hours until the lungs begin to clear up.

Eli Lilly & Company supplies Ampoules No. 28 Camphor in Oil. Each ampoule contains 10 mils of solution representing 36 grains of camphor. The fine reputation for quality enjoyed by Eli Lilly & Company, known as THE Ampoule House, is assurance to the physician that in specifying Lilly Ampoules there can be no doubt of the therapeutic actvity of the preparation.

Our readers are referred to Eli Lilly & Company for further information on this or other subjects pertaining to ampoule medication. It is said that this concern offers the most complete line of American made ampdules to be found in this country.

